# Supplementary material for: Heterogenous Model of Temozolomide Resistance in Glioblastoma Reveals Phenotypic Shifts in Drug Response and Migratory Potential
Source: Adv Nanobiomed Res. Author manuscript; Available in PMC 2026 Apr 1. (PMC13038291; doi:10.1002/anbr.202500244)
Supplement: Supplementary Information [file NIHMS2155779-supplement-Supplementary_Information.pdf]

Supporting Information

Heterogenous Model of Temozolomide Resistance in Glioblastoma Reveals Phenotypic Shifts in Drug Response and Migratory Potential

Victoria A. Kriuchkovskaia[1,2], Ela K. Eames[1], Sydney A. McKee [2,4], Paul J. Hergenrother[2,3,4], Rebecca B. Riggins[5], Brendan A.C. Harley[1,2,3]

[1] Dept. Chemical and Biomolecular Engineering
[2] Carl R. Woese Institute for Genomic Biology
[3] Cancer Center at Illinois
[4] Dept. of Chemistry
University of Illinois Urbana-Champaign
Urbana, IL 61801, USA.

[5] Dept. of Oncology, Lombardi Comprehensive Cancer Center,
Georgetown University Medical Center
Washington, DC 20057, USA.

Corresponding Author:
B.A.C. Harley
Dept. of Chemical and Biomolecular Engineering
Cancer Center at Illinois
Carl R. Woese Institute for Genomic Biology
University of Illinois at Urbana-Champaign
110 Roger Adams Laboratory
600 S. Mathews Ave.
Urbana, IL 61801
Phone: (217) 244-7112
Fax: (217) 333-5052
e-mail: bharley@illinois.edu

Supplemental Figures

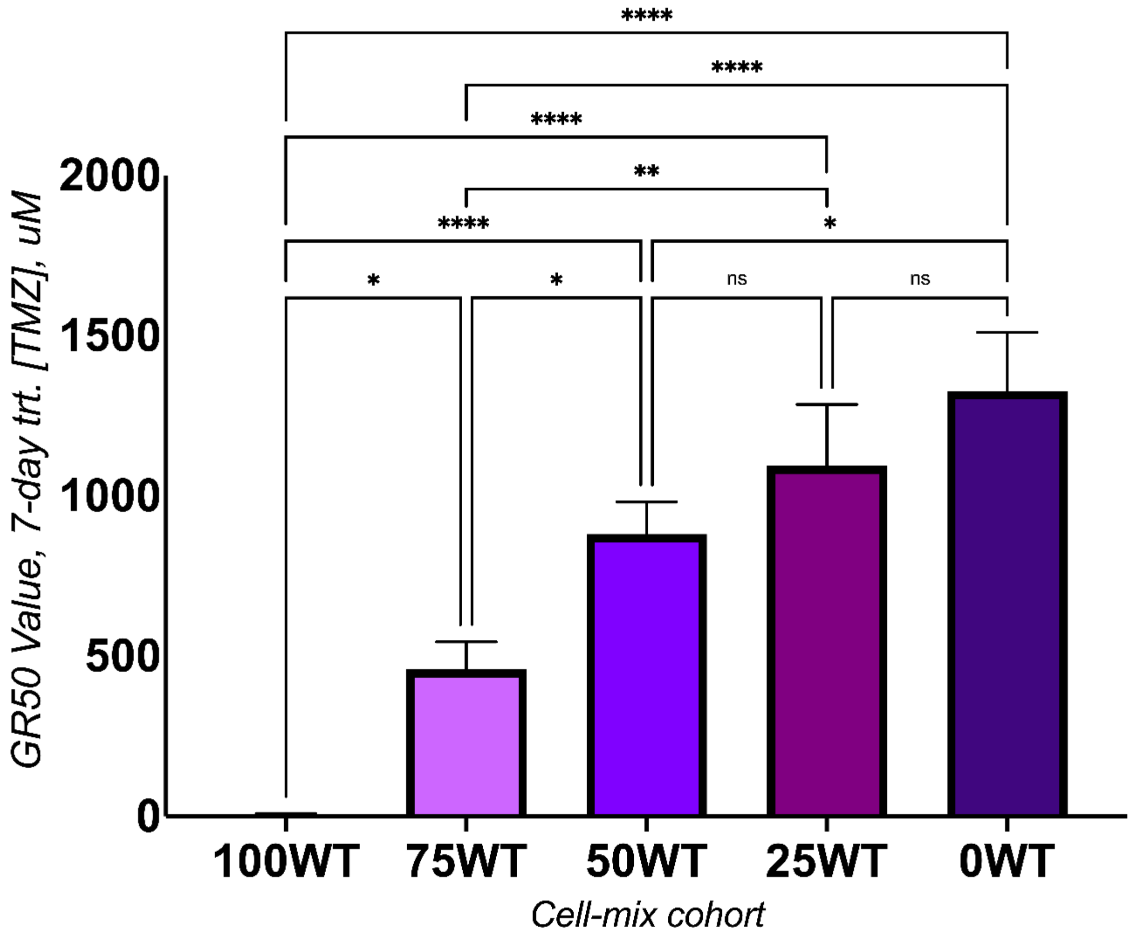

Figure S1. Calculated TMZ GR50 values for each group 7-days post single-dose TMZ treatment. GR values showed a progressive increase in resistance from the 42WT-only group (6.6 µM) to the 42TMZres-only group (1327 µM). GR50 values for mixed cell populations were intermediate, with the 75WT group at 450 µM, 50WT at 881 µM, and 25WT at 1094 µM. *: $p < 0.05$. **: $p < 0.01$. ***: $p < 0.001$. Figure was created in GraphPad Prism.

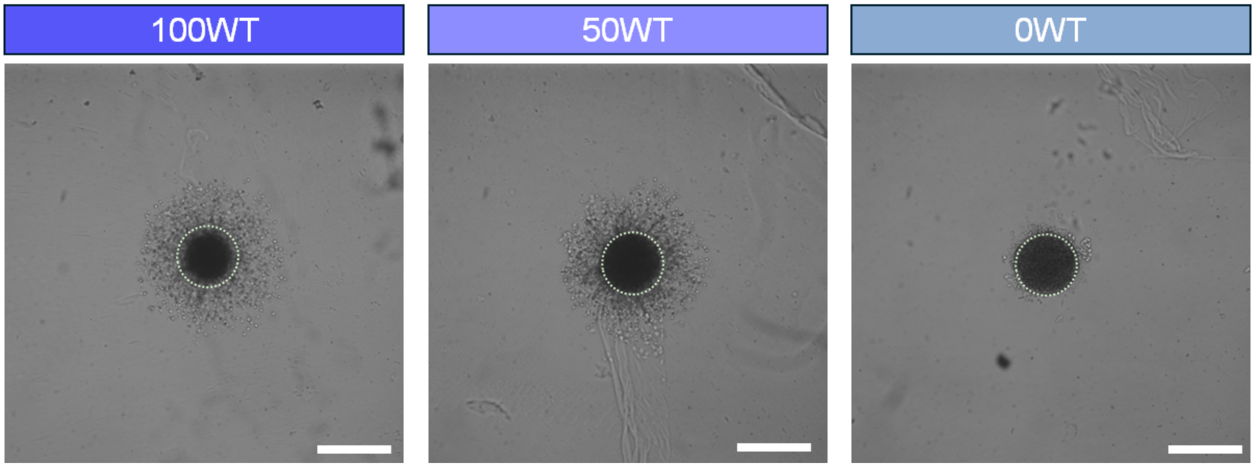

Figure S2. Representative images of 0WT, 50WT, and 100WT GBM spheroids encapsulated into GelMA matrix and treated with 0uM TMZ (control). Brightfield images capture radial spread of GBM cells into the surrounding GelMA hydrogel matrix from initial spheroid (dashed line). Scale bar: 500 µm.
